# Cancer-protective effect of a synbiotic combination between *Lactobacillus gasseri* 505 and a *Cudrania tricuspidata* leaf extract on colitis-associated colorectal cancer

**DOI:** 10.1080/19490976.2020.1785803

**Published:** 2020-07-14

**Authors:** Nam Su Oh, Ji Young Lee, You-Tae Kim, Sae Hun Kim, Ju-Hoon Lee

**Affiliations:** a Department of Food and Biotechnology, Korea University, Sejong, South Korea; b Department of Biotechnology, College of Life Sciences and Biotechnology, Korea University, Seoul, South Korea; c Department of Food Science and Biotechnology, Graduate School of Biotechnology, Kyung Hee University, Yongin, South Korea

**Keywords:** synbiotics, immune modulation, apoptosis, tight junction, colorectal cancer, microbiome

## Abstract

Previously, a synbiotic combination of probiotic *Lactobacillus gasseri* 505 (LG) and a new prebiotic, *Cudrania tricuspidata* leaf extract (CT) in fermented milk, designated FCT, showed an *in vitro* immunomodulatory effect and antioxidant activity. Although synbiotic combination might have cancer-protective effects, these activities have not been fully validated *in vivo*. Ten-week treatment of LG, CT, or FCT to azoxymethane (AOM)/dextran sodium sulfate (DSS)-induced colitis-associated colorectal cancer (CAC) mouse model reduced both the incidence of colonic tumors and damage to the colonic mucosa effectively, suggesting a cancer-protective effect. To understand these, biomarkers associated with inflammation, colon barrier, apoptosis, and cancer cell proliferation were monitored in AOM/DSS group versus LG/CT/FCT groups. A synbiotic combination (FCT) down-regulated pro-inflammatory cytokines (TNF-α, IFN-γ, IL-1β, and IL-6) and inflammation-associated enzymes (iNOS and COX-2), and up-regulated anti-inflammatory cytokines (IL-4 and IL-10). In addition, colon barrier experiment revealed that biomarkers of mucus layer (MUC-2 and TFF3) and tight junction (occludin and ZO-1) were up-regulated. Subsequent apoptosis experiment showed that pro-apoptotic factors (p53, p21, and Bax) were up-regulated and anti-apoptotic factors (Bcl-2 and Bcl-xL) were down-regulated. Furthermore, comparative metagenome analysis of gut microbiota revealed that *Staphylococcus* decreased but *Lactobacillus, Bifidobacterium*, and *Akkermansia* increased, supporting their protective effects, accompanied by increased short-chain fatty acids (SCFAs). Taken together, the FCT administration showed cancer-protective effects by reducing the risk of colitis-associated colon cancer via regulation of inflammation, carcinogenesis, and compositional change of gut microbiota. Consequently, the synbiotic combination (FCT) could be a novel potential health-protective natural agent against CAC.

## Introduction

Colorectal cancer (CRC) is the third most commonly diagnosed cancer but has the second highest mortality rate.^1^ Chronic inflammation has been suggested to be one of the hallmarks of cancer development.^2^ CRC is known to be strongly associated with chronic inflammation, which can be present from the early stage of tumor onset.^3^ Thus, inflammatory bowel disease (IBD), including ulcerative colitis and Crohn′s disease (CD), can increase the risk of developing a type of CRC referred to as colitis-associated cancer (CAC).^3^ Epidemiological studies have reported that IBD patients exhibit a two- to eightfold higher CRC risk, and the incidence of CRC can be effectively ameliorated by anti-inflammatory medications.^4,5^ Although anti-inflammatory medications (e.g., non-steroidal anti-inflammatory drugs (NSAID) and selective COX-2 inhibitors) can reduce IBD-related CRC formation, there are limitations for their long-term use because of life-threatening side effects.^6^ The demand for more effective and safer natural agents to prevent colon cancer has, therefore, increased. Consequently, it is essential to explore alternative approaches to managing IBD and help prevent inflammation-associated colon cancer.

The main cause of IBD may be associated with a combination of inflammation factors, including inherited genetic host susceptibility, composition of host gut microbiota, unbalanced immune response, and oxidative stress.^7^ However, their mechanisms remain unknown. Among them, oxidative stress may cause neutrophils to overproduce colonic oxidants, including reactive oxygen species (ROS), which may contribute to intestinal tissue damage by mucosal inflammation, causing IBD.^8^ To substantiate this, a previous report showed that superoxide dismutase overexpression in *Lactobacillus gasseri* revealed an anti-inflammatory effect on, and recovery of, colonic tissue in an Interleukin-10 (IL-10)-deficient mouse model of colitis, alleviating IBD symptoms.^9^ Therefore, it may be important to develop safe and natural agents harboring antioxidant activity for further scientific evaluation as a novel approach against IBD and even CRC. In particular, probiotics, prebiotics, and their combinational synbiotics may be a new therapeutic approach against IBD as safe and natural agents.^7^ For example, combining resistant starch and *Bifidobacterium animalis* subsp. *lactis* showed a protective effect in an azoxymethane (AOM)-induced rodent model of CRC.^10^ A recent report suggests that proper modulation of gut microbiota, by ingesting specific probiotics, may help prevent tumor formation.^11^ Furthermore, ingesting prebiotics, which is defined as host non-digestible food ingredients, are fermented by specific beneficial intestinal microbes. They are known to be beneficial to the host by stimulating probiotic bacteria growth, suggesting that they may change human gut microbiota composition. Therefore, they were suggested to help specific probiotics for CRC prevention.^12^ Based on this, it is necessary to find proper probiotics and/or prebiotics with strong antioxidant activity and to confirm their activities with *in vitro* and *in vivo* experiments.

*L. gasseri* 505 (LG) was preliminarily isolated from a healthy infant fecal sample but little grew in milk.^13^ In addition, a *Cudrania tricuspidata* leaf extract (CT), which is a new plant-based prebiotic source, successfully enhanced the specific growth of *L. gasseri* 505 in milk, showing pH lowering effect.^13,14^ Interestingly, the fermented milk improved antioxidant activity through a synbiotic milk fermentation by a combination of *L. gasseri* and CT.^15^ A subsequent experiment revealed that fermenting CT and milk by *L. gasseri* 505 produced specific phenolic compounds and bioactive peptides with antioxidant activities. Their structures and amino acid sequences were identified using LC-MS/MS and MALDI-TOF/MS, respectively.^13^ To verify the immune response’s modulation activity, RAW 264.7 macrophages were treated with fermented milk with synbiotics of *L. gasseri* 505 and CT; several cytokines were evaluated, showing *in vitro* immunomodulatory effects.^16^ However, the probiotics, prebiotics, and synbiotic combinations’ preventive effects against IBD-associated inflammation have not yet been explored in *in vivo* models.

In this study, we aimed to investigate the cancer-protective effect of fermented milk with a synbiotic combination of *L. gasseri* 505 and CT (designated FCT) in a CRC mouse model induced with AOM/dextran sodium sulfate (DSS). We examined several colon barrier function markers, inflammation, apoptosis, the cell cycle, and the regulatory pathways involved in colon cancer development to understand the possible action mechanisms. Furthermore, metagenome analysis, using high-throughput microbial 16 S rRNA gene sequencing, was used to better understand the effects of FCT on the relationship between microbial metabolism and colon cancer.

## Results

### General responses of colon

**Figure 1.**
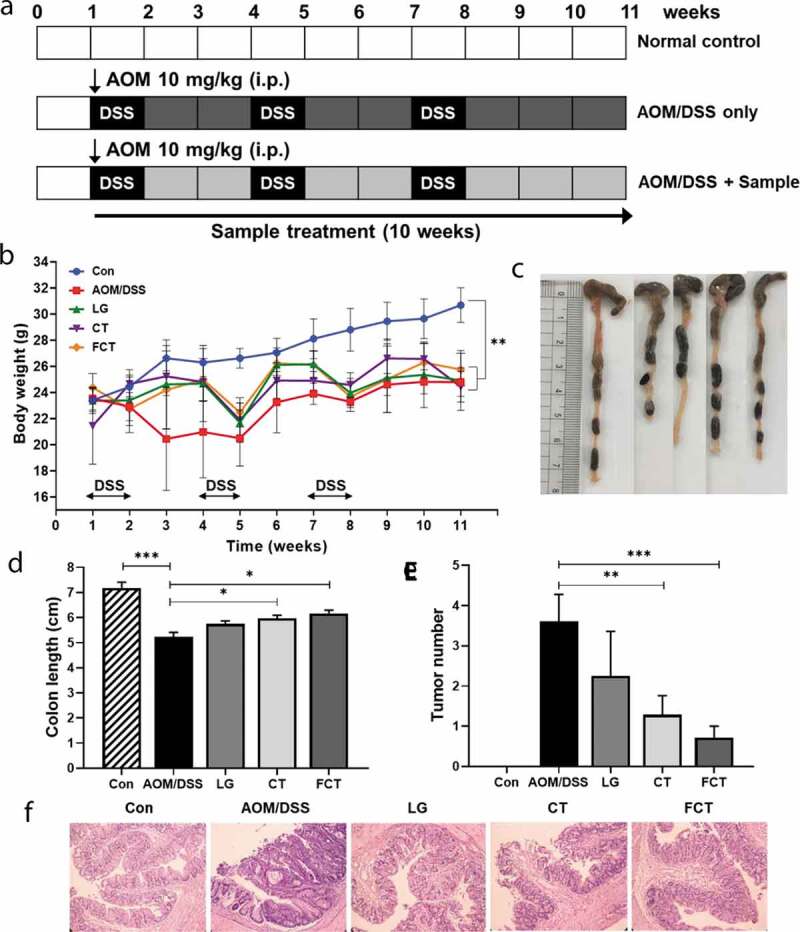
Tumor development with AOM/DSS in mice and recovery of the AOM/DSS-induced colitis-associated colorectal cancer (CAC) mouse model with *L. gasseri* 505 (LG), *C. tricuspidata* leaf extract (CT), and fermented CT by *L. gasseri* 505 (FCT). (a) Experimental procedure for development of the AOM/DSS-induced CAC model and sample (LG, CT, or FCT) administration. Mice were given a single intraperitoneal (i.p.) injection of AOM (10 mg/kg) and then received 2.5% DSS in the drinking water for one week, followed by two weeks of regular drinking water for recovery; this treatment cycle was repeated three times. The sample (LG, CT, or FCT) was administered orally in the CAC mice for 10 weeks. (b) Change of average body weight (g) of the mice in the groups; control mouse (Con), AOM-DSS-induced CAC mouse (AOM/DSS), LG, CT, and FCT. (c) A photograph for measuring and comparing colon lengths of the groups at Week 11. (d) A graph of average colon lengths in the groups at Week 11. (e) Occurrence of colon tumors in the groups. The data present the mean ± standard deviation (SD). Asterisks denote significance vs. AOM/DSS group by one-way ANOVA (*p < .05, **p < .01, ***p < .001). (f) H&E staining of representative histological sections of colons from the groups (200 × magnification).

FCT’s preventive effect on colon carcinogenesis was examined using an AOM/DSS-induced CAC model. DSS administration reduced the weight of mice in all groups (Figure 1(b)). However, the LG, CT, and FCT groups showed a protective effect on weight loss from DSS treatment compared with the AOM/DSS control group. Among them, the FCT group showed a slightly higher protective effect on weight loss at Week 11. Furthermore, the AOM/DSS control group had notable shortening of the colon compared with the untreated normal control group. However, LG, CT, and FCT significantly prevented AOM/DSS-induced colonic shortening (Figure 1(c–d) and Figure S1(a)). All mice in the AOM/DSS group had colon tumors, but treatment with LG, CT, or FCT suppressed neoplastic development. Among them, the FCT group showed the highest suppressive effect in colon tumor cells (Figure 1(e) and Figure S1(b)). To clarify this, the H&E-stained colon tissues were histologically examined to observe colonic inflammation and mucosal injury in mice (Figure 1(f)). Tissue sections from representative areas of the colons of normal control group mice showed intact surface epithelia, intestinal glands, stroma, and submucosa, whereas the AOM/DSS control group mice showed evidence of distorted crypt epithelia and extensive mucosal damage. In contrast, the dysplasia and structural disruption were reduced in the LG and CT groups compared to the AOM/DSS control group, whereas treatment with the FCT group showed evidence of relatively well-preserved crypt structures and further improvements in histological features (Figure S1(c)). Dysplasia and adenocarcinoma development were rarely observed in the FCT group.

### Regulation of inflammatory responses

**Figure 2.**
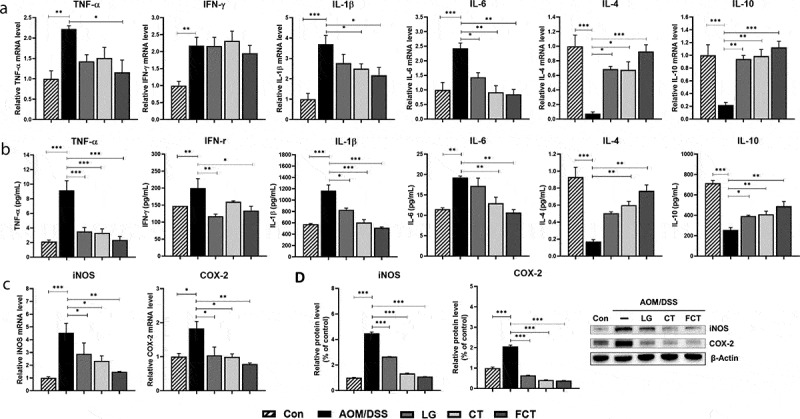
Immune response of the mouse colon by treatment of AOM/DSS and administration of LG, CT, and FCT. (a) mRNA quantification of pro-inflammatory cytokines (TNF-α, IFN-γ, IL-1β, and IL-6) and anti-inflammatory cytokines (IL-4 and IL-10) using Real-Time RT-PCR. (b) Determination of protein productions of the inflammatory cytokines using ELISA. (c) mRNA quantification of iNOS and COX-2 using Real-Time RT-PCR. (d) Determination of protein productions of iNOS and COX-2 using Western blot analysis. The mRNA level was normalized with an mRNA level of GAPDH, and the protein production was normalized with β-actin. The data present the mean ± standard deviation (SD). Asterisks denote significance vs. AOM/DSS group by one-way ANOVA (*p < .05, **p < .01, ***p < .001). The abbreviations of Con, AOM/DSS, LG, CT, and FCT represent the control mice, AOM-DSS-induced CAC mice, *L. gasseri* 505, *C. tricuspidata* leaf extract, and fermented CT by *L. gasseri* 505, respectively.

Inflammatory molecules are involved in the various processes that occur in colitis-associated colon carcinogenesis.^5^ To elucidate the inflammatory response in AOM/DSS-induced CAC mice, various pro- and anti-inflammatory cytokines were measured in colon tissues in mRNA and protein level. The mRNA expressions of pro-inflammatory cytokines, such as tumor necrosis factor-α (TNF-α), Interferon-γ (IFN-γ), IL-1β, and IL-6, increased significantly in the AOM/DSS control group compared to the normal control group (Figure 2(a)). However, their mRNA expressions were gradually reduced in the LG, CT, and FCT groups. In particular, mRNA expressions in the FCT group were highly suppressed up to a level similar to the normal control group. In contrast with this result, the mRNA expressions of anti-inflammatory cytokines, such as IL-4 and IL-10, decreased significantly in the AOM-DSS control group (Figure 2(a)). However, their mRNA expressions gradually increased in the LG and CT groups and were highest in the FCT group. This inflammatory response suggests reduced inflammation of AOM-DSS-damaged tissue cells, via the stabilization of inflammatory response to the AOM/DSS-untreated group, compared to the AOM/DSS-untreated normal control group. Quantification of specific cytokines using ELISA showed the same patterns in regulation of production and secretion of pro- and anti-inflammatory cytokines via inflammatory response in AOM-DSS-damaged tissue cells, supporting this result (Figure 2(b)).

The inducible nitrogen oxide synthase (iNOS) producing nitric oxide (NO) is stimulated by pro-inflammatory cytokines, suggesting that iNOS induction is associated with host immunity, and the produced high level of NO could provide host cell toxicity and damage with its free radical.^17^ In addition, inhibition of COX-2 (cyclooxygenase-2) is related to suppressed inflammation and pain relief.^18^ While mRNA expressions of iNOS and COX-2 in colon tissue were the highest in the AOM/DSS control group, treatments of LG, CT, and FCT gradually reduced their mRNA expressions (Figure 2(c)). Interestingly, FCT treatment of AOM/DSS-induced CAC mice reduced mRNA expressions, which were similar to those of normal control group, suggesting that FCT treatment could reduce colon tissue cell damage and suppress inflammation. The finding was substantiated with subsequent experiments for protein quantification using Western blot analysis, which showed similar patterns of protein production levels of iNOS and COX-2 in all groups. This result may be also associated with reduced pro-inflammatory cytokines after FCT treatment (Figure 2(b, d)).

### Recovery of colon barrier in AOM/DSS-induced CAC mice

**Figure 3.**
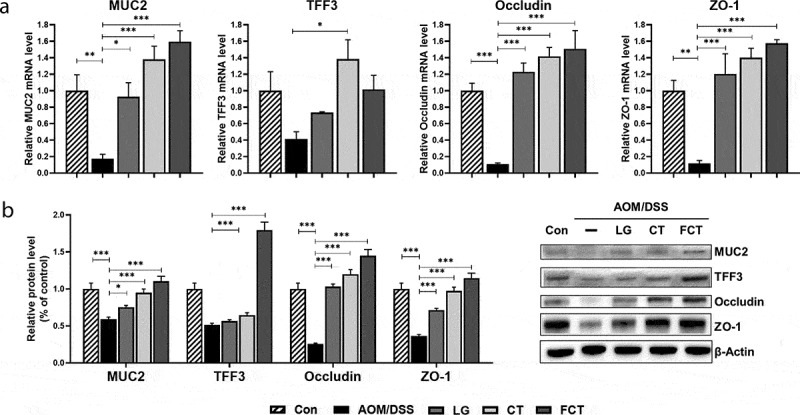
Recovery of mucus- (MUC2 and TFF3) and colon barrier-associated (occludin and ZO-1) proteins from the colons of the AOM/DSS-induced CAC mice by *L. gasseri* 505 (LG), *C. tricuspidata* leaf extract (CT), and fermented CT by *L. gasseri* 505 (FCT). The abbreviations of Con and AOM/DSS represent the control mice and AOM-DSS-induced CAC mice, respectively. (a) mRNA quantification of MUC2, TFF3, occludin, and ZO-1 using Real-Time RT-PCR with respect to the mRNA level of GAPDH for normalization. (b) Determination of the protein productions using Western blot analysis compared with β-actin production for normalization. The data present the mean ± standard deviation (SD). Asterisks denote significance vs. AOM/DSS group by one-way ANOVA (*p < .05, **p < .01, ***p < .001).

Tight junctions are known to have a vital role in maintaining cell-to-cell integrity, and their loss is important in the pathophysiology of a variety of gastrointestinal disorders such as IBD, irritable bowel syndrome, and even CRC.^19^ Therefore, mucin-associated proteins (MUC2, oligomeric mucus gel-forming protein; TFF3, mucin-associated peptide trefoil factor 3) and tight-junction structural proteins (occludin and zonula occludens-1 (ZO-1)) were measured in mRNA and protein levels. Their mRNA expressions were significantly suppressed in the AOM/DSS control group compared to the normal control group. However, their mRNA expression levels rapidly recovered and were even higher than the normal control group, suggesting the mucosal layer and tight junction recovered from damage with AOM/DSS (Figure 3(a)). In particular, the FCT group showed the highest mRNA expression level in MUC2, occludin, and ZO-1. The subsequent protein quantification showed the same results (Figure 3(b)). This result suggests that FCT treatment protects the epithelial barrier by recovery or even enhancement of mucus- and tight junction-associated proteins in AOM/DSS-induced CAC mice.

### Enhancement of apoptosis and suppression of tumor cell proliferation

**Figure 4.**
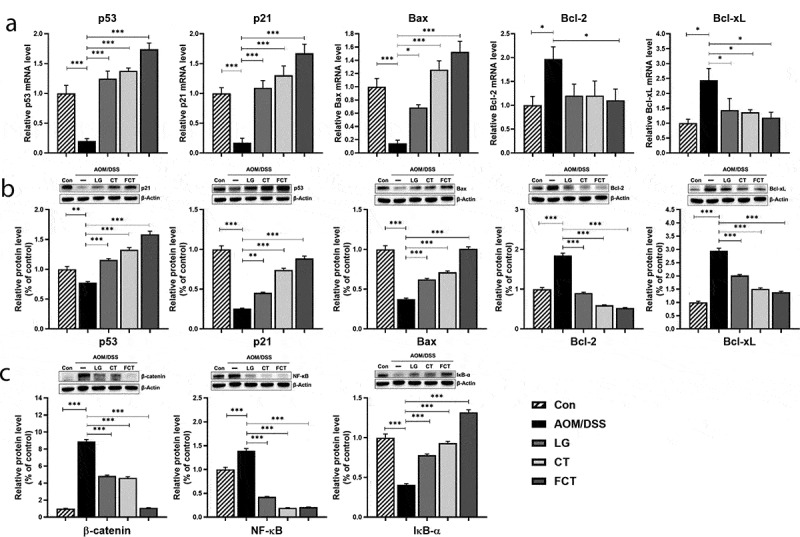
Effects of dietary *L. gasseri* 505 (LG), *C. tricuspidata* leaf extract (CT), and fermented CT by *L. gasseri* 505 (FCT) on the colon tissues of the AOM/DSS-induced CAC mice by determination of pro-apoptosis (p53, p21, and Bax), anti-apoptosis markers (Bcl-2 and Bcl-xL), and tumor cell proliferation markers (β-catenin, NF-κB, and IκBα). (a) mRNA quantification of the apoptosis markers using Real-Time RT-PCR. (b) Determination of the protein productions of the apoptosis markers using Western blot analysis. (c) Determination of the protein productions of the tumor cell proliferation markers using Western blot analysis. The mRNA level was normalized with the mRNA level of GAPDH, and the protein production was normalized with β-actin. The data present the mean ± standard deviation (SD). Asterisks denote significance vs. AOM/DSS group by one-way ANOVA (*p < .05, **p < .01, ***p < .001).

Uncontrolled proliferation and avoidance of apoptosis are considered common events during colon carcinogenesis. To elucidate apoptosis regulation in the tumorigenesis of colon tissue using LG, CT, and FCT, changes in mRNA expressions and protein production in apoptosis-associated and anti-apoptosis markers were monitored. The mRNA expressions of apoptosis-associated markers, such as p53, p21, and Bax, were suppressed in the AOM/DSS control group but gradually increased in the LG, CT, and FCT groups (Figure 4(a)). In particular, FCT treatment significantly increased mRNA expressions at the highest level, which was much higher than those of the normal control group. However, mRNA expressions of anti-apoptosis markers, such as Bcl-2 and Bcl-xL, gradually reduced in the LG, CT, and FCT groups, suggesting enhanced apoptosis for colon carcinogenesis suppression (Figure 4(a)). Their Western blot analysis results showed the same protein production patterns in the LG, CT, and FCT groups (Figure 4(b)).

In addition to the apoptosis-associated response, mRNA expression and protein production of tumor cell proliferation-related markers, such as β-catenin, NF-κB, and IκB-α, were measured in the colon tissues. β-catenin is a key signaling molecule for colon carcinogenesis, and NF-κB is a transcription factor that induces gene expression for tumor cell proliferation. However, IκB-α blocks specific DNA binding activity of the NF-κB transcription factor.^20^ Treatment of LG, CT, or FCT to AOM/DSS-induced CAC mice revealed that the mRNA expression and protein production of β-catenin and NF-κB reduced significantly compared to the AOM/DSS control group (Figure 4(c)). However, those of IκB-α gradually increased as expected. Therefore, FCT treatment may suppress tumor cell proliferation or colon carcinogenesis by inducing cell cycle arrest and apoptosis in the tumorigenesis of colon tissue of the mice.

### Changes of microbial community structure

**Figure 5.**
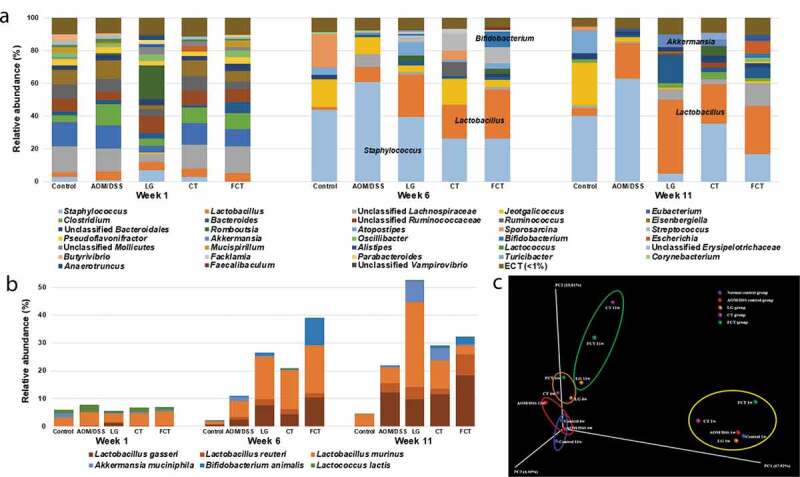
Effects on changes of gut microbiota composition in mice by treatment of AOM/DSS and administration of *L. gasseri* 505 (LG), *C. tricuspidata* leaf extract (CT), and fermented CT by *L. gasseri* 505 (FCT). (a) Compositional analysis of mouse gut microbiota in the groups at Weeks 1, 6, and 11 using 16 S rRNA full sequencing. (b) Compositional analysis of representative beneficial bacteria in mouse gut, *Lactobacillus, Bifidobacterium, Akkermansia*, and *Lactococcus* at Week 1, 6, and 11. (c) Principle coordinates analysis (PCoA) plot of the groups at Weeks 1, 6, and 11 with the weighted Unifrac distance matrix.

Administration of LG, CT, and FCT was predicted to change gut microbiota composition in AOM/DSS-induced CAC mice. To evaluate this, fecal samples were collected from Weeks 1, 6, and 11, and the collected samples were used for metagenome analysis of gut microbiota composition. During the first collection of fecal samples in Week 1, no AOM/DSS was treated and no LG/CT/FCT were administrated. Metagenome analysis of all groups showed they had similar gut microbiota composition in Week 1 (Figure 5(a)).

However, subsequent metagenome analysis in Week 6 showed that the gut microbiota composition in all groups was quite different from Week 1, suggesting that these changes may be due to feed change with AIN-76, AOM/DSS treatment, and LG/CT/FCT administrations. In particular, compositional change of gut microbiota in the normal control group between Weeks 1 and 6 may be due to only feed change because there was no AOM/DSS treatment and no LG/CT/FCT administration, suggesting that feed change may have an important factor in the change of gut microbiota composition in Week 6. In addition to feed change, AOM/DSS treatment and LG/CT/FCT administration showed additional composition changes in other groups (Figure 5(a)). Previously, it was shown that AOM/DSS treatment induced loss of body weight, shortening of colon length, and even CAC by gut inflammation (Figure 1). Based on this, AOM/DSS treatment in Week 6 showed significant increments of *Staphylococcus* in the AOM/DSS control group (Figure 5(a)). Further, 16 S rRNA full sequence analysis showed that the major species of this *Staphylococcus* are *S. lentus* and *S. sciuri*, which may be associated with inflammatory diseases.^21^ However, LG, CT, and FCT administrations gradually reduced *Staphylococcus* composition, suggesting that they may reduce gut inflammation in Week 6. This result is consistent with previous results regarding pro-inflammatory cytokines, substantiating reduced inflammation by FCT (Figure 2). In addition, LG, CT, and FCT administrations increased the compositions of *Lactobacillus, Akkermansia*, and *Bifidobacterium* in these groups. Subsequent 16 S rRNA full sequence analysis in LG, CT, and FCT groups revealed that major species of this *Lactobacillus* are *L. gasseri, L. reuteri*, and *L. murinus*, which may be associated with anti-inflammation (Figure 5(b)).^9,22,23^ Previous administration of *L. gasseri* to AOM/DSS-induced CAC mice reduced pro-inflammatory cytokines in LG and FCT groups (Figure 2). Therefore, compositional changes of gut microbiota from Week 1 to Week 6 by LG, CT, and FCT administrations suggest reduced gut inflammation. Furthermore, compositional increment of *A. muciniphila* and *B. animalis* in the gut may be beneficial for gut microbiota recovery in AOM/DSS-induced CAC mice (Figure 5(b)).

While bacterial compositions of the normal control group and the AOM/DSS control group were very similar between Week 6 and Week 11, those of other groups of LG and FCT changed, especially those of *Staphylococcus* and *Lactobacillus* (Figure 5(a)). *Staphylococcus* compositions in the LG and FCT groups of Week 11 were much lower than those of Week 6, suggesting that daily administration of *L. gasseri* to only these two groups may inhibit *Staphylococcus* growth in the gut. The high proportions of *Lactobacillus* in the bacterial composition of these groups support this hypothesis (Figure 5(a)). However, *Staphylococcus* composition in the CT group did not change, probably because CT treatment without *L. gasseri* administration may be limited to inhibiting *Staphylococcus* growth via propagation of *L. gasseri* (Figure 5(a)). It is noteworthy that *L. gasseri* composition increased gradually in the sampling periods of Weeks 1, 6, and 11 (Figure 5(b)). Previous inflammation and CAC-associated results in this study suggested that the population of *L. gasseri* with CT supplementation may be a key factor for anti-inflammation and even CAC (Figure 1, Figure 2). Therefore, compositional change of gut microbiota by FCT administration, such as increments of anti-inflammatory *Lactobacillus* as well as reduction of pro-inflammatory *Staphylococcus* in FCT group, may play an important role in anti-CAC effects. Additional Principal coordinates analysis (PCoA) plot analysis also supports the hypothesis that change in gut microbiota composition for anti-inflammation and anti-CAC effects may be due to feed change and LG/CT/FCT administration (Figure 5(c)). Consequently, FCT administration to AOM/DSS-induced CAC mice may be the most effective treatment for anti-inflammation and even CAC.

### Modulation of fecal SCFA concentrations

**Figure 6.**
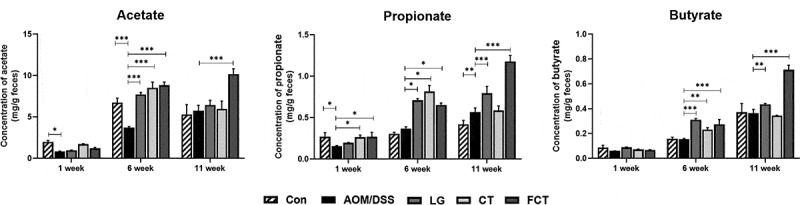
Measurement of short-chain fatty acids (SCFAs), acetate, propionate, and butyrate, in the collected fecal samples using GC-MS at Weeks 1, 6, and 11. The data present the mean ± standard deviation (SD). Asterisks denote significance vs. AOM/DSS group by one-way ANOVA (*p < .05, **p < .01, ***p < .001). The abbreviations of Con, AOM/DSS, LG, CT, and FCT represent the control mice, AOM-DSS-induced CAC mice, *L. gasseri* 505, *C. tricuspidata* leaf extract, and fermented CT by *L. gasseri* 505, respectively.

SCFA is produced by intestinal microbiota, especially lactic acid bacteria as well as bifidobacteria, which play an important role in maintaining healthy gut microbiota and preventing intestinal disease.^24^ Administration of LG/CT/FCT to AOM/DSS-induced CAC mice previously showed increments of *Lactobacillus, Bifidobacterium*, and *Akkermansia* in this study, which have been known as SCFA-producing bacteria.^25^ To verify this, subsequent SCFA analysis revealed that the administration of LG/CT/FCT increases acetate, propionate, and butyrate in their groups in Weeks 6 and 11 (Figure 6). Among them, FCT administration to AOM/DSS-induced CAC mice in Week 11 showed maximum production. Because it was previously reported that SCFA production inhibits the growth of some pathogens in the gut,^26^ LG, CT, and especially FCT may suppress the growth of specific pathogens in the mouse model. Previous metagenome analysis of gut microbiota result showed reduced *Staphylococcus* in gut microbiota composition (Figure 5(a)), suggesting that the relative increment of those specific gut bacteria and their SCFA productions may be responsible for *Staphylococcus* suppression. Consequently, results of the metagenome analysis and SCFA analysis confirmed that FCT administration is the best for stimulating specific gut bacteria growth and their SCFA production in AOM/DSS-induced CAC mice; therefore, FCT might play an important role in suppressing inflammation-associated bacteria.

## Discussion

While the main causes of inflammatory diseases such as IBD and CRC have been suggested to include genetic disorders, unbalanced immune response, and gut microbiota dysbiosis, the oxidative stress by ROS overproduction may be one of the important factors in the development of inflammatory diseases.^27,30^ To reduce this oxidative stress, previous papers suggested that probiotics and/or prebiotics might be effective via modulation of gut microbiota.^30,31^ According to this suggestion, an *L. gasseri* 505 (LG) was selected as a new probiotic strain, due to its antioxidant activity, and a *C. tricuspidata* leaf extract (CT) was used as a natural prebiotic source, which is specific for *L. gasseri*. Milk fermentation with LG and CT revealed pH a lowering effect and specific growth stimulation of LG by CT.^15^ In particular, this fermented milk with the synbiotic combination (FCT) showed antioxidant activity with bioactive peptides from β-casein and phenolic compounds from CT.^13^ In addition, subsequent cytokine assay with FCT showed a synbiotic immunomodulatory activity. Consequently, it was previously confirmed through *in vitro* assays that FCT provides two major activities, antioxidant activity by secondary metabolites after fermentation and immunomodulatory activity by immune response via inflammatory cytokines.^16^ Those FCT activities may help reduce inflammation, IBD, and even CRC. Previous recent papers also suggested another mechanism on intestinal repair by anti-oxidative and cytoprotective activities that *Lactobacillus* can mediate ROS production by NADPH oxidase in. This ROS activates Nrf2 xenobiotic pathway and this pathway mediates beneficial anti-oxidative and cytoprotective effects to drive intestinal repair.^32,33^ Although this new mechanism was not evaluated in this study, it may be necessary to investigate this hypothesis in near future.

To evaluate FCT’s protective effects *in vivo*, it was administrated to AOM/DSS-induced CAC mice. AOM and DSS are genotoxic and non-genotoxic colonic carcinogens, respectively. Their combination led to colitis-related carcinogenesis development through inflammation of colon cells.^34^ In addition, subsequent optimization of the combination revealed that continuous administration of low concentrations of DSS with a dose of AOM results in powerful tumor-promoting activity.^35^ This method was used to develop an AOM/DSS-induced CAC mouse model in this study. Administration of LG/CT/FCT to AOM/DSS-induced CAC mice during 10 weeks led to gradual increases in body weight and colon length, as well decreased the number of tumor cells, compared with the AOM/DSS control group, suggesting the recovery of damaged colon cells by LG/CT/FCT. The histology results support this recovery action by LG/CT/FCT. This recovery action of damaged colon cells following AOM/DSS treatment has been also reported in other publications.^36,38^

Colonic inflammation has been suggested to be an important factor for developing IBD and even CRC.^39^ Therefore, its prevention may be a good strategy for suppressing IBD and CRC in the colon. Homeostasis of Th1 and Th2 cells is a key for regulating inflammation. Pro-inflammatory cytokines, IFN-γ and IL-1β, stimulate maturation of naïve CD4 + T cells to Th1 cells. Then, Th1 cells increase the production of IFN-γ and TNF-α. This IFN-γ suppresses Th2 cell proliferation. Because of this interaction, stimulation of Th1 cells and suppression of Th2 cells increases the Th1/Th2 ratio, probably related to inflammation. On the contrary, IL-4 stimulates the maturation of naïve CD4 + T cells to Th2 cells. Matured Th2 cells increase IL-4 production and suppress Th1 cell proliferation by IL-4 and IL-10. This stimulation of Th2 cell and suppression of Th1 cells reduces the Th1/Th2 ratio, probably alleviating inflammation. Therefore, to restore the Th1/Th2 balance, it is necessary to reduce pro-inflammatory cytokines, such as IFN- γ, TNF-α, IL-1β, and IL-6, as well as to increase anti-inflammatory cytokines, such as IL-4 and IL-10.^40^ In addition to the reduction of Th1/Th2 ratio as an anti-inflammatory cytokine, IL-4 drives switch of Ig class to IgG and IgE. IgG is the main antibody and it protects the body from infections by various pathogens and neutralizes toxins. However, IgE plays an essential role in Type I hypersensitivity, associated with various allergic diseases including asthma and atopy. Therefore, it was suggested that overproduction of IL-4 may be associated with atopy via IgE production.^41^ Furthermore, IL-4 suppresses Th1 cell proliferation, suggesting that it also has anti-inflammatory action in the Th1-driven inflammation. In addition to the balance of Th1/Th2 ratio, AOM/DSS treatment causes colon tissue damage and it induces cytokine productions by neutrophil, monocyte, and macrophage.^42^ After colon tissue damage, like T and B cells, these cells produce cytokines including TNF-α, IL-1β, and IL-6, causing inflammatory cell infiltration. Therefore, total cytokines are produced from T cell, B cell, and these three other cells. In this study, total cytokines from colon tissue were measured and these pro-inflammatory and anti-inflammatory cytokines were compared to understand the inflammatory responses to AOM/DSS, LG, CT, or FCT treatment.

In this study, AOM/DSS treatment of normal mice increases pro-inflammatory cytokines and decreases anti-inflammatory cytokines (Figure 2(a–b)). Previous observations of shortened colon lengths and tumor cell proliferation may be associated with this (Figure 1(c–e)). However, administration of LG, CT, and FCT to AOM/DSS-induced CAC mice showed opposite directions in production of those cytokines (Figure 2(a–b)), which was probably associated with recovered colon length and reduced the number of tumor cells (Figure 1(c–e)), suggesting the anti-inflammatory effect of LG, CT, and FCT in the mouse model. Based on this result, AOM/DSS treatment provoked colonic carcinogenesis in mice, probably due to breakdown of the balanced Th1/Th2 ratio, but administration of LG/CT/FCT ameliorates this symptom by restoring homeostasis of the Th1/Th2 ratio. Recent papers reported the same results in the mouse models with colitis.^43,45^

Inducible nitric oxide synthase (iNOS) is an enzyme that produces nitrogen oxide (NO) from L-arginine. This NO reacts with superoxide (O_2_^−^), which produces peroxynitrite (ONOO^−^).^46^ This intermediate compound acts as oxidative stress to damage intestinal epithelial cells via DNA cleavage, lipid peroxidation, thiol oxidation, and carbohydrate degradation, causing inflammation.^47,49^ Therefore, iNOS regulation is important for controlling inflammation in the intestinal epithelial cells. To regulate mRNA expression and its protein production of an iNOS gene, pro-inflammatory cytokines, such as IL1-α, IFN-γ, and TNF-α, induced mRNA expression of the iNOS gene and its protein production. However, anti-inflammatory cytokines, such as IL-4 and IL-13, inhibit mRNA expression as well as protein production, suggesting that inflammatory cytokines are involved in regulating iNOS production.^50,51^ In addition, it is well known that cyclooxygenase-2 (COX-2) converts arachidonic acid to prostaglandin (PG), which is a mediator for pain, inflammation, and fever.^52^ In particular, NSAIDs including aspirin, inhibit COX-2’s enzyme activity to reduce the bioconversion of arachidonic acid to PG as painkillers.^53^ Therefore, inhibiting COX-2’s mRNA expression and protein production is an important key to prevent epithelial cell inflammation. In this study, while mRNA expressions as well as protein productions of iNOS and COX-2 were induced by AOM/DSS treatment, administration of LG/CT/FCT gradually inhibited their mRNA expressions as well as protein productions in AOM/DSS-induced CAC mice, suggesting that LG, CT, and FCT could reduce colonic inflammation by inhibiting iNOS and COX-2 (Figure 2(c–d)). The associations of iNOS and COX-2 with epithelial cell inflammation have been confirmed experimentally in other studies, even though they work in different mechanisms for inflammation.^54,55^

The mucus layer and the epithelial tight junction are important for regulating mucosal permeability for ions, nutrients, and water as the intestinal barrier.^56,57^ This tight junction consists of a multi-protein complex forming a selectively permeable seal between adjacent epithelial cells. This interacts with intestinal microbiota, food components, intestinal fluids, mucosal layer, intrinsic nerves, and even immune system components for its regulation.^58,62^ Oligomeric mucus gel-forming protein (Mucin-2, MUC-2) and mucin-associated peptide trefoil factor 3 (TFF3) have been known as main component proteins of the mucin layer, related to the epithelial tight junction.^63,64^ The multi-protein complex is composed of claudin, occludin, tricellulin, and junction adhesion molecule as transmembrane proteins. Their intracellular parts interact with cytoplasmic peripheral membrane proteins such as zonular occludens (ZO-1, −2, and −3) and cingulin.^65,68^ These proteins interact with F-actin and myosin II in epithelial cells to control contraction and barrier loss of tight junction.^69^ In particular, this barrier loss has been suggested to be responsible for various intestinal diseases. The barrier dysfunction is regulated by pro-inflammatory cytokines such as TNF-α, IFN-γ, and IL-1β. This regulatory mechanism consists of inhibiting mRNA expression of occludin gene by IFN-γ and TNF-α and enhancing mRNA expression of the myosin light chain kinase (MLCK) gene by TNF-α and IL-1β.^70,71^ Through this mechanism, reducing component proteins of tight junction, and contraction of myosin II in epithelial cells by phosphorylation of myosin light chain protein with MLCK, cause the barrier loss.^72^ Therefore, TNF-α and MLCK are thought to be therapeutic targets for barrier dysfunction of epithelial tight junction.^70^ In this study, treatment of AOM/DSS to normal mice caused inflammation in the epithelial cells, releasing pro-inflammatory cytokines such as TNF-α, IFN-γ, and IL-1β. These cytokines are responsible for the reduction of component proteins of tight-junction systems such as MUC-2 and TFF3 in the mucus layer and reduction of occludin and ZO-1 in the tight junction, suggesting barrier loss. Subsequent administration of LG/CT/FCT to these damaged mice with barrier loss showed rapid increases in mRNA and protein levels to overcome this barrier loss by recovery of mucus layer and tight junction, suggesting that LG, CT, and FCT can recover the AOM/DSS-induced CAC mice (Figure 3(a–b)).

Apoptosis is a programmed cell death event caused by cell changes, such as blebbing, morphological change, cell shrinkage, chromatin condensation, apoptotic DNA fragmentation, and mRNA decay.^73^ While cancer cells avoid the apoptosis for carcinogenesis, apoptosis may be a process for suppression and even death of cancer cells.^74^ The tumor suppressor protein p53 plays an important role in triggering apoptosis by up-regulating the pro-apoptotic BCL2-associated X protein (Bax).^75^ This Bax protein induces opening of the mitochondrial voltage-dependent anion channel, indicating the induction of mitochondrial outer membrane permeability. This induced permeability helps release cytochrome C and other pro-apoptotic proteins, as well as activating caspases. Finally, they enhance p53-mediated apoptosis in cancer cells.^76^ However, B-cell lymphoma 2 (Bcl-2) in mitochondrial outer membrane promotes cell survival and inhibits the action of pro-apoptotic proteins to regulate apoptosis.^77^ In addition, B-cell lymphoma-extra-large (Bcl-xL) is a transmembrane molecule in mitochondria, which reduces mitochondrial outer membrane permeability to block the release of cytochrome C and pro-apoptotic proteins.^78^ Therefore, Bcl-2 family proteins contribute to cell survival by anti-apoptosis. In addition, the cyclin-dependent kinase inhibitor 1 (p21) protein is induced by p53 protein, and it inhibits the activities of cyclin/CDK complexes. In particular, p21 binding to CDK2 suppresses cancer cell proliferation and arrests them in the G1 phase.^79^ Therefore, p53 protein induces cancer cell apoptosis by Bax, cytochrome C, and caspases, and inhibits cancer cell proliferation by inducing p21. In this study, AOM/DSS treatment of normal mice inhibited p53, p21, and Bax significantly, but this treatment increased Bcl-2 and Bcl-xL production, suggesting suppression of apoptosis and enhancement of cell proliferation, probably for carcinogenesis. However, subsequent administration of LG/CT/FCT revealed the opposite result, suggesting anti-carcinogenesis (Figure 4(a–b)). Therefore, this result suggests that LG, CT, and FCT can reduce intestinal carcinogenesis caused by AOM/DSS treatment in mice.

The nuclear factor κ-light-chain-enhancer of activated B cells (NF-κB) is a heterodimer protein consisting of RelA (p65) and p50 proteins. It regulates the transcription of specific genes associated with cell proliferation for survival.^80^ Under normal conditions, the NF-κB/IκBα complex is in cytoplasm, and IκBα inhibits NF-κB activity. Once extracellular signals activate IκB kinase (IKK), IκBα is phosphorylated by IKK, activating NF-κB. The activated NF-κB moves into the nucleus and binds to a response element (RE), specific DNA sequences in the chromosome. Binding of RE-bound NF-κB with a coactivator and RNA polymerase initiates specific gene transcription, associated with cell proliferation.^81^ In the case of cancer cells, NF-κB is constitutively active, indicating that it expresses genes for cancer cell proliferation and protecting them from apoptosis. Therefore, suppression of NF-κB and activation of IκBα are important factors for preventing cancer cell proliferation.^82^ In the Wnt pathway, Wnt signaling initiates β-catenin phosphorylation by casein kinase 1 (CK1) and glycogen synthase kinase 3 beta (GSK3β). This Wnt signaling stimulates β-catenin accumulation in the cytoplasm; the accumulated β-catenin is then translocated into the nucleus. It promotes the transcription of specific genes involved in the production of cancer proteins.^83,84^ In particular, the adenomatous polyposis coli and β-catenin were suggested to be the key genes involved in CRC development.^85^ Therefore, β-catenin has been suggested as a therapeutic target for suppressing cancer cell proliferation.^86^ In this study, AOM/DSS treatment increased β-catenin and NF-κB but reduced IκBα. However, LG/CT/FCT administrations significantly suppress β-catenin and NF-κB production but promote IκBα production (Figure 4(c–d)). Consequently, inhibiting β-catenin and NF-κB and promoting IκBα by LG, CT, and FCT may suppress carcinogenesis and cancer cell proliferation by inhibiting cancer-associated gene transcription.

Cancer development has been suggested to be connected with the intestinal gut microbiota.^87^ Previous papers reported that chronic inflammation and intestinal epithelium disruption might be associated with infection from pathogens and dysbiosis in gut microbiota.^88,89^ However, these diseases can be recovered to a normal state via treatment with probiotics and/or prebiotics.^90,91^ This microbiome study showed that changes in gut microbiota composition were caused by feed change, AOM/DSS treatment, and LG/CT/FCT administrations. Feed change with AIN-76 modified overall gut microbiota composition between Weeks 1 and 6 (Figure 5(a)). In addition, AOM/DSS treatment of normal mice for the development of AOM/DSS-induced CAC mouse model increased *Staphylococcus* composition in Weeks 6 and 11 (Figure 5(a)). In these periods, administration of LG, CT, and FCT to this mouse model reduced this genus but increased *Lactobacillus, Bifidobacterium*, and *Akkermansia*, suggesting suppression of pro-inflammatory *Staphylococcus* and promotion of anti-inflammatory beneficial bacteria. This result is consistent with a previous observation of colonic cancer development in the AOM/DSS group and colonic cancer suppression in the LG/CT/FCT groups. In addition, an immune response assay of pro- and anti-inflammatory cytokines, and an apoptosis assay of several pro- and anti-apoptotic factors, substantiate this. Furthermore, gradual increases in SCFAs during the test periods suggest the recovery of AOM/DSS-damaged gut microbiota in AOM/DSS-induced CAC mice by LG, CT, and FCT (Figure 6).

**Figure 7.**
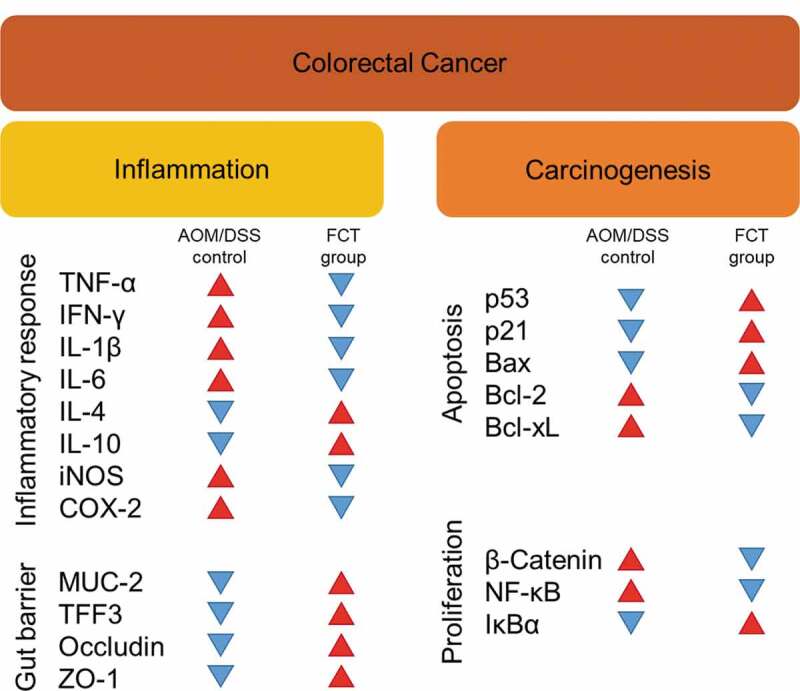
The result summary of AOM/DSS treatment and FCT administration in the mouse model showing that FCT administration to AOM/DSS-induced CAC mouse has regulatory effects on reduction of inflammation, recovery of colon barrier, and enhancement of tumor cell apoptosis, indicating the anticancer effect.

This study was performed *in vivo* to extend our understanding of synbiotics’ anticancer effects (LG, CT, or FCT) in the CAC mice model. Colon observation after AOM/DSS treatment showed loss of body weight, reduced colon length, development of tumor cells, and destruction of mucosal epithelial layers (Figure 1(b–f)). However, administration of synbiotics revealed the recovery of damaged gut by AOM/DSS. To understand these phenomena, many biomarkers associated with inflammation, colon barrier, apoptosis, and cancer cell proliferation were monitored in the AOM/DSS group and the LG/CT/FCT groups. The immune response experiment showed that pro-inflammatory cytokines (TNF-α, IFN-γ, IL-1β, and IL-6) and inflammation-associated enzymes (iNOS and COX-2) were down-regulated, and anti-inflammatory cytokines (IL-4 and IL-10) were up-regulated, by FCT administration in AOM/DSS-induced CAC mice. In addition, a colon barrier experiment revealed that all biomarkers of mucus layer (MUC-2 and TFF3) and tight junction (occludin and ZO-1) were up-regulated by FCT administration. An apoptosis experiment showed that pro-apoptotic factors (p53, p21, and Bax) were up-regulated and anti-apoptotic factors (Bcl-2 and Bcl-xL) were down-regulated in the FCT group. In addition, a cancer cell proliferation experiment revealed that transcription regulators (β-catenin and NF-κB) were down-regulated but the transcription inhibitor (IκBα) was up-regulated in the FCT group. These results are summarized in Figure 7. Furthermore, comparative metagenome analysis of gut microbiota and subsequent SCFA analysis were also consistent with these results. Consequently, all results in this study with FCT indicate that they can reduce inflammation and carcinogenesis in gut-damaged mice by AOM/DSS treatment and even recover dysbiosis of gut microbiota. Accordingly, these results suggest that FCT has an anticancer effect in CRC through the regulation of several colon cancer-related signaling markers and gut microbiota, and it could be a natural preventive agent against inflammation-associated colon tumorigenesis.

## Materials and Methods

### Preparation and characterization of fermented milk

*C. tricuspidata* leaves were obtained from the local market (Sunchang, Jeollabuk-Do, Korea). *L. gasseri* 505 (LG) was selected as the probiotic strain because it has clear probiotic effects.^13^ The extract of *C. tricuspidata* leaves (CT) and the synbiotic combination as fermented milk containing CT by *L. gasseri* 505 (FCT) were prepared using our previous methods.^15^

### Animal model of AOM/DSS-induced colorectal cancer

Male C57BL/6 mice (8-week old) were purchased from Samtaco Bio Korea (Osan, Korea). The animals were maintained at 22 ± 2°C and 55 ± 5% relative humidity with a 12-h light/dark cycle. Feed (AIN-76; DooYeol Biotech, Seoul, Korea) and water were supplied *ad libitum*. After a seven-day adaptation period, mice were randomly allocated to five groups (n = 10/group): an untreated normal control group (only PBS treatment), an AOM/DSS control group (only AOM/DSS treatment), an LG group (AOM/DSS treatment and *L. gasseri* 505 (10⁸ CFU/kg/day)), a CT group (AOM/DSS treatment and *C. tricuspidata* leaf extract-supplemented milk (1.5 g/kg/day)), and an FCT group (AOM/DSS treatment and *C. tricuspidata* leaf extract-supplemented milk fermented with *L. gasseri* 505 (1.5 g/kg/day)). To induce colon carcinogenesis using AOM and DSS,^5^ mice were intraperitoneally (i.p.) injected with 10 mg/kg AOM (Sigma-Aldrich) at the start point of Week 1 and then received 2.5% DSS (Sigma-Aldrich) in their drinking water every day for 1 week, followed by regular drinking water for next 2 weeks to allow for recovery. While AOM administration was performed only one time during Week 1, DSS administration was repeated three times for whole test periods (Weeks 1–2, 4–5, and 7–8) (Figure 1(a)). The feeding study was performed for 10 weeks after a one-week adaptation period. Body weights were measured every week during the study. The mice were euthanized at Week 11, and their colons were excised. After the colons’ lengths were measured, they were washed with PBS and cut open longitudinally. The gross tumors were quantified. The colon was severed longitudinally into two pieces. One-half of the colon was fixed in 10% neutral-buffered formalin (Sigma-Aldrich) and used for hematoxylin and eosin (H&E) staining. The other half was stored in liquid nitrogen for later extraction and real-time PCR, enzyme-linked immunosorbent assays (ELISA), and Western blot analyses. All experiments involving mice were approved by the institutional animal care and use committee of Korea University (Seoul, Korea; approval number KUIACUC-2016-182) and conducted in accordance with Care and Use of Laboratory Animals guidelines.

### Histopathological analysis

Fixed colon segments were embedded in paraffin using standard procedures, and 5-μm sections were stained with H&E.^92^ Colon tissues were examined and imaged using an Olympus CKX41 microscope (Tokyo, Japan) equipped with a Canon EOS 600D camera (Tokyo, Japan).

### Detection of cytokine levels

For the ELISAs for TNF-α, IFN-γ, IL-1β, IL-6, IL-4, and IL-10, colon tissue samples were homogenized in standard RIPA buffer (Invitrogen) supplemented with a protease inhibitor cocktail (Roche). The lysates were centrifuged at 13,000 × *g* at 4°C for 30 min, and the supernatants were collected. Colonic cytokine levels were determined using the appropriate ELISA kits (ELISA MAX Deluxe Sets; BioLegend) according to the manufacturer’s recommendations. Optical densities were measured at 450 nm, subtracting the background measured at 570 nm.

### Western blot analysis

The total protein concentration of the colon homogenate supernatant was determined with a BCA protein assay kit (Thermo Scientific). Protein samples (10 μg) were fractionated by electrophoresis on 12% SDS-polyacrylamide gels and transferred to a PVDF membrane, using the Trans-Blot Turbo™ Transfer System (Bio-Rad). The membranes were blocked with 5% skimmed milk in wash buffer (0.1% Tween-20 in TBS; TBST) for 1 h, and then incubated with primary antibody overnight at 4°C. Following this, the blots were incubated with the appropriate horseradish peroxidase (HRP)-conjugated secondary antibody for 1 h at room temperature. The antibodies used in this study are listed in Table S1. After washing, the membranes were visualized, using an enhanced chemiluminescence (ECL) substrate (Bio-Rad), and imaged using the Bio-Rad ChemiDoc MP imaging system. Western blot bands were quantified using ImageJ software (National Institutes of Health).

### Total RNA extraction and quantitative real-time PCR

Total RNA was extracted from colon tissues, using TRIzol reagent (Invitrogen) following the manufacturer’s instructions. RNA was quantified using a NanoDrop 2000 spectrophotometer (Thermo Scientific) prior to cDNA synthesis using a first-strand cDNA Synthesis Kit (LeGene). Quantitative real-time PCR was performed with the StepOnePlus Real-Time PCR System (Applied Biosystems), using the KAPA SYBR FAST qPCR Kit (Kapa Biosystems). Primer sequences are listed in Table S2. Real-time PCR data were analyzed by the 2^−ΔΔCt^ method,^93^ normalizing readings to that of GAPDH.

### Microbial community analysis using PacBio system

To elucidate the gut microbiota composition, PCR amplification of whole-length 16 S rRNA gene was performed with total fecal DNA and 27 F/1492 R universal primer set.^94^ Gel-purified 16 S rRNA PCR products were randomly sequenced by ChunLab (Seoul, Korea) using the PacBio system with its standard manufacturer’s protocol. After sequencing a quality check of raw reads, primer regions in the reads were trimmed with ChunLab’s in-house program at a similarity cut off 0.8. The EzTaxon database is used for taxonomic assignment using BLAST algorism, and bacterial community and statistical analyses were performed using QIIME 1.9.1.^95,96^

### Fecal short-chain fatty acid (SCFA) analysis

Feces were collected immediately following defecation, transferred to cryotubes, and snap-frozen in liquid nitrogen. The frozen samples were ground, and the fecal SCFA content was determined by gas chromatography coupled to a mass spectrometer (GC-MS), as previously reported.^97^

### Statistical analysis

All data are expressed as means ± SD. Statistical significance was assessed by Duncan’s multiple range test. SAS software version 9.2 (SAS Institute Inc.) was used to perform all statistical tests. *P* < .05 was considered statistically significant. Pearson’s correlation was analyzed to investigate the relationships between relative abundance of fecal microbiota and cancer-associated markers.

## Supplementary Material

Supplemental Material

## References

[1] Bray F, Ferlay J, Soerjomataram I, Siegel RL, Torre LA, Jemal A. Global cancer statistics 2018: GLOBOCAN estimates of incidence and mortality worldwide for 36 cancers in 185 countries. CA Cancer J Clin. 2018;68:394–20. doi:10.3322/caac.21492. 30207593

[2] Hanahan D, Weinberg RA. Hallmarks of cancer: the next generation. Cell. 2011;144:646–674. doi:10.1016/j.cell.2011.02.013. 21376230

[3] Chumanevich AA, Poudyal D, Cui X, Davis T, Wood PA, Smith CD, Hofseth LJ. Suppression of colitis-driven colon cancer in mice by a novel small molecule inhibitor of sphingosine kinase. Carcinogenesis. 2010;31:1787–1793. doi:10.1093/carcin/bgq158. 20688834 PMC2981458

[4] van Staa TP, Card T, Logan R, Leufkens H. 5-Aminosalicylate use and colorectal cancer risk in inflammatory bowel disease: a large epidemiological study. Gut. 2005;54:1573–1578. doi:10.1136/gut.2005.070896. 15994215 PMC1774734

[5] B Vendramini-Costa D, E Carvalho J. Molecular link mechanisms between inflammation and cancer. Curr Pharm Des. 2012;18:3831–3852. doi:10.2174/138161212802083707. 22632748

[6] Wang D, DuBois RN. The role of anti-inflammatory drugs in colorectal cancer. Annu Rev Med. 2013;64:131–144. doi:10.1146/annurev-med-112211-154330. 23020877

[7] Sartor RB. Therapeutic manipulation of the enteric microflora in inflammatory bowel diseases: antibiotics, probiotics, and prebiotics. Gastroenterology. 2004;126:1620–1633. doi:10.1053/j.gastro.2004.03.024. 15168372

[8] Keshavarzian A, Banan A, Farhadi A, Komanduri S, Mutlu E, Zhang Y, Fields J. Increases in free radicals and cytoskeletal protein oxidation and nitration in the colon of patients with inflammatory bowel disease. Gut. 2003;52:720–728. doi:10.1136/gut.52.5.720. 12692059 PMC1773652

[9] Carroll IM, Andrus JM, Bruno-Bárcena JM, Klaenhammer TR, Hassan HM, Threadgill DS. Anti-inflammatory properties of *Lactobacillus gasseri* expressing manganese superoxide dismutase using the interleukin 10-deficient mouse model of colitis. Am J Physiol Gastrointest Liver Physiol. 2007;293:G729–G38. doi:10.1152/ajpgi.00132.2007. 17640978

[10] Le Leu RK, Hu Y, Brown IL, Woodman RJ, Young GP. Synbiotic intervention of *Bifidobacterium lactis* and resistant starch protects against colorectal cancer development in rats. Carcinogenesis. 2010;31:246–251. doi:10.1093/carcin/bgp197. 19696163

[11] Genaro SC, de Souza Reis LSL, Reis SK, Socca EAR, Fávaro WJ. Probiotic supplementation attenuates the aggressiveness of chemically induced colorectal tumor in rats. Life Sci. 2019;237:116895. doi:10.1016/j.lfs.2019.116895. 31610204

[12] Rong J, Liu S, Hu C, Liu C. Single probiotic supplement suppresses colitis‐associated colorectal tumorigenesis by modulating inflammatory development and microbial homeostasis. J Gastroenterol Hepatol. 2019;34:1182–1192. doi:10.1111/jgh.14516. 30357910

[13] Oh NS, Lee JY, Oh S, Joung JY, Kim SG, Shin YK, Lee K-W, Kim SH, Kim Y. Improved functionality of fermented milk is mediated by the synbiotic interaction between *Cudrania tricuspidata* leaf extract and *Lactobacillus gasseri* strains. Appl Microbiol Biotechnol. 2016;100:5919–5932. doi:10.1007/s00253-016-7414-y. 26996626

[14] Oh NS, Lee JY, Joung JY, Kim KS, Shin YK, Lee K-W, Kim SH, Oh S, Kim Y. Microbiological characterization and functionality of set-type yogurt fermented with potential prebiotic substrates *Cudrania tricuspidata* and *Morus alba* L. Leaf Extracts J Dairy Sci. 2016;99:6014–6025. doi:10.3168/jds.2015-10814. 27236762

[15] Oh NS, Lee JY, Kim Y. The growth kinetics and metabolic and antioxidant activities of the functional synbiotic combination of *Lactobacillus gasseri* 505 and *Cudrania tricuspidata* leaf extract. Appl Microbiol Biotechnol. 2016;100:10095–10106. doi:10.1007/s00253-016-7863-3. 27796437

[16] Lee JY, Kim SG, Shin YK, Oh NS. Immunomodulatory effects of fermented milk based on synbiotic interaction between *Cudrania tricuspidata* leaf extract and *Lactobacillus gasseri* 505. J Milk Sci Biotechnol. 2018;36:39–48. doi:10.22424/jmsb.2018.36.1.39.

[17] Sies H, De Groot H. Role of reactive oxygen species in cell toxicity. Toxicol Lett. 1992;64:547–551. doi:10.1016/0378-4274(92)90230-H. 1335181

[18] Samad TA, Moore KA, Sapirstein A, Billet S, Allchorne A, Poole S, Bonventre JV, Woolf CJ. Interleukin-1β-mediated induction of Cox-2 in the CNS contributes to inflammatory pain hypersensitivity. Nature. 2001;410:471–475. doi:10.1038/35068566. 11260714

[19] Martin TA, Jiang WG. Loss of tight junction barrier function and its role in cancer metastasis. Biochim Biophys Acta Biomembr. 2009;1788:872–891. doi:10.1016/j.bbamem.2008.11.005. 19059202

[20] Jacobs MD, Harrison SC. Structure of an IκBα/NF-κB complex. Cell. 1998;95:749–758. doi:10.1016/S0092-8674(00)81698-0. 9865693

[21] Stepanović S, Dakić I, Martel A, Vaneechoutte M, Morrison D, Shittu A, Ježek P, Decostere A, Devriese LA, Haesebrouck F. A comparative evaluation of phenotypic and molecular methods in the identification of members of the *Staphylococcus sciuri* group. Syst Appl Microbiol. 2005;28:353–357. doi:10.1016/j.syapm.2005.02.001. 15997708

[22] Liu Y, Fatheree NY, Mangalat N, Rhoads JM. Human-derived probiotic *Lactobacillus reuteri* strains differentially reduce intestinal inflammation. Am J Physiol Gastrointest Liver Physiol. 2010;299:G1087–G96. doi:10.1152/ajpgi.00124.2010. 20798357 PMC2993169

[23] Pan F, Zhang L, Li M, Hu Y, Zeng B, Yuan H, Zhao L, Zhang C. Predominant gut *Lactobacillus murinus* strain mediates anti-inflammaging effects in calorie-restricted mice. Microbiome. 2018;6:54. doi:10.1186/s40168-018-0440-5. 29562943 PMC5863386

[24] Tan J, McKenzie C, Potamitis M, Thorburn AN, Mackay CR, Macia L. The role of short-chain fatty acids in health and disease. In: Macia L, editor. Advances in immunology. Vol. 121. Cambridge (MA): Elsevier; 2014. p. 91–119. 10.1016/B978-0-12-800100-4.00003-9 24388214

[25] LeBlanc JG, Chain F, Martín R, Bermúdez-Humarán LG, Courau S, Langella P. Beneficial effects on host energy metabolism of short-chain fatty acids and vitamins produced by commensal and probiotic bacteria. Microb Cell Fact. 2017;16:79. doi:10.1186/s12934-017-0691-z. 28482838 PMC5423028

[26] Sun Y, O’Riordan MX. Regulation of bacterial pathogenesis by intestinal short-chain fatty acids. In: O’Riordan MX, editor. Advances in applied microbiology. Vol. 85. Cambridge (MA): Elsevier; 2013. p. 93–118. 10.1016/B978-0-12-407672-3.00003-4 PMC4029053 23942149

[27] Jurk D, Wilson C, Passos JF, Oakley F, Correia-Melo C, Greaves L, Saretzki G, Fox C, Lawless C, Anderson R. Chronic inflammation induces telomere dysfunction and accelerates ageing in mice. Nat Commun. 2014;5:4172. doi:10.1038/ncomms5172. PMC4090717 24960204

[28] Josefowicz SZ, Niec RE, Kim HY, Treuting P, Chinen T, Zheng Y, Umetsu DT, Rudensky AY. Extrathymically generated regulatory T cells control mucosal TH 2 inflammation. Nature. 2012;482:395–399. doi:10.1038/nature10772. 22318520 PMC3485072

[29] Gevers D, Kugathasan S, Denson LA, Vázquez-Baeza Y, Van Treuren W, Ren B, Schwager E, Knights D, Song SJ, Yassour M. The treatment-naive microbiome in new-onset Crohn’s disease. Cell Host Microbe. 2014;15:382–392. doi:10.1016/j.chom.2014.02.005. 24629344 PMC4059512

[30] Ballini A, Santacroce L, Cantore S, Bottalico L, Dipalma G, Topi S, Saini R, De Vito D, Inchingolo F. Probiotics efficacy on oxidative stress values in inflammatory bowel disease: a randomized double-blinded placebo-controlled pilot study. Endocr Metab Immune Disord Drug Targets. 2019;19:373–381. doi:10.2174/1871530319666181221150352. 30574857

[31] D’Souza A, Fordjour L, Ahmad A, Cai C, Kumar D, Valencia G, Aranda JV, Beharry KD. Effects of probiotics, prebiotics, and synbiotics on messenger RNA expression of caveolin-1, NOS, and genes regulating oxidative stress in the terminal ileum of formula-fed neonatal rats. Pediatr Res. 2010;67:526–531. doi:10.1203/PDR.0b013e3181d4ff2b. 20101198

[32] Jones RM, Desai C, Darby TM, Luo L, Wolfarth AA, Scharer CD, Ardita CS, Reedy AR, Keebaugh ES, Neish AS. Lactobacilli modulate epithelial cytoprotection through the Nrf2 pathway. Cell Rep. 2015;12:1217–1225. doi:10.1016/j.celrep.2015.07.042. 26279578 PMC4640184

[33] Matthews JD, Owens JA, Naudin CR, Saeedi BJ, Alam A, Reedy AR, Hinrichs BH, Sumagin R, Neish AS, Jones RM. Neutrophil-derived reactive oxygen orchestrates epithelial cell signaling events during intestinal repair. Am J Pathol. 2019;189:2221–2232. doi:10.1016/j.ajpath.2019.07.017. 31472109 PMC6892184

[34] Parang B, Barrett CW, Williams CS. AOM/DSS model of colitis-associated cancer. In: Williams CS editor. Gastrointestinal physiology and diseases. New York (NY): Springer; 2016. p. 297–307. 10.1007/978-1-4939-3603-8_26 PMC5035391 27246042

[35] De Robertis M, Massi E, Poeta ML, Carotti S, Morini S, Cecchetelli L, Signori E, Fazio VM. The AOM/DSS murine model for the study of colon carcinogenesis: from pathways to diagnosis and therapy studies. J Carcinog. 2011;10:9. doi:10.4103/1477-3163.78279. 21483655 PMC3072657

[36] Santiago C, Pagán B, Isidro AA, Appleyard CB. Prolonged chronic inflammation progresses to dysplasia in a novel rat model of colitis-associated colon cancer. Cancer Res. 2007;67:10766–10773. doi:10.1158/0008-5472.CAN-07-1418. 18006820

[37] Chung I-C, OuYang C-N, Yuan S-N, Lin H-C, Huang K-Y, Wu P-S, Liu C-Y, Tsai K-J, Loi L-K, Chen Y-J. Pretreatment with a heat-killed probiotic modulates the NLRP3 inflammasome and attenuates colitis-associated colorectal cancer in mice. Nutrients. 2019;11:516. doi:10.3390/nu11030516. PMC6471765 30823406

[38] Jeong J-K, Chang H-K, Park K-Y. Doenjang prepared with mixed starter cultures attenuates azoxymethane and dextran sulfate sodium-induced colitis-associated colon carcinogenesis in mice. J Carcinog. 2014;13:9. doi:10.4103/1477-3163.137699. 10.4103/1477-3163.137699 PMC4141357 25191137

[39] Grivennikov SI. Inflammation and colorectal cancer: colitis-associated neoplasia. In: Grivennikov SI editor. Seminars in Immunopathology. New York (NY): Springer; 2013. p. 229–244. 10.1007/s00281-012-0352-6 PMC3568220 23161445

[40] Zhu J, Paul WE. CD4 T cells: fates, functions, and faults. Blood. 2008;112:1557–1569. doi:10.1182/blood-2008-05-078154. 18725574 PMC2518872

[41] Hershey GK, Friedrich MF, Esswein LA, Thomas ML, Chatila TA. The association of atopy with a gain-of-function mutation in the alpha subunit of the interleukin-4 receptor. N Engl J Med. 1997;337:1720–1725. doi:10.1056/NEJM199712113372403. 9392697

[42] Kanda Y, Osaki M, Okada F. Chemopreventive strategies for inflammation-related carcinogenesis: current status and future direction. Int J Mol Sci. 2017;18:867. doi:10.3390/ijms18040867. 10.3390/ijms18040867 PMC5412448 28422073

[43] Saito Y, Hinoi T, Adachi T, Miguchi M, Niitsu H, Kochi M, Sada H, Sotomaru Y, Sakamoto N, Sentani K. Synbiotics suppress colitis-induced tumorigenesis in a colon-specific cancer mouse model. PLoS One. 2019;14:e0216393. doi:10.1371/journal.pone.0216393. 31242213 PMC6594584

[44] Shinde T, Perera AP, Vemuri R, Gondalia SV, Karpe AV, Beale DJ, Shastri S, Southam B, Eri R, Stanley R. Synbiotic supplementation containing whole plant sugar cane fibre and probiotic spores potentiates protective synergistic effects in mouse model of IBD. Nutrients. 2019;11:818. doi:10.3390/nu11040818. PMC6521199 30979002

[45] Yan S, Yang B, Zhao J, Zhao J, Stanton C, Ross RP, Zhang H, Chen W. A ropy exopolysaccharide producing strain *Bifidobacterium longum* subsp. *longum* YS108R alleviates DSS-induced colitis by maintenance of the mucosal barrier and gut microbiota modulation. Food Funct. 2019;10:1595–1608. doi:10.1039/C9FO00014C. 30806428

[46] Guzik TJ, West NE, Pillai R, Taggart DP, Channon KM. Nitric oxide modulates superoxide release and peroxynitrite formation in human blood vessels. Hypertension. 2002;39:1088–1094. doi:10.1161/01.HYP.0000018041.48432.B5. 12052847

[47] Stamler JS, Jia L, Eu JP, McMahon TJ, Demchenko IT, Bonaventura J, Gernert K, Piantadosi CA. Blood flow regulation by S-nitrosohemoglobin in the physiological oxygen gradient. Science. 1997;276:2034–2037. doi:10.1126/science.276.5321.2034. 9197264

[48] Wink DA, Cook JA, Kim SY, Vodovotz Y, Pacelli R, Krishna MC, Russo A, Mitchell JB, Jourd’heuil D, Miles AM. Superoxide modulates the oxidation and nitrosation of thiols by nitric oxide-derived reactive intermediates. Chemical aspects involved in the balance between oxidative and nitrosative stress. J Biol Chem. 1997;272:11147–11151. doi:10.1074/jbc.272.17.11147. 9111012

[49] Kolios G, Valatas V, Ward SG. Nitric oxide in inflammatory bowel disease: a universal messenger in an unsolved puzzle. Immunology. 2004;113:427–437. doi:10.1111/j.1365-2567.2004.01984.x. 15554920 PMC1782592

[50] Kolios G, Brown Z, Robson RL, Robertson DA, Westwick J. Inducible nitric oxide synthase activity and expression in a human colonic epithelial cell line, HT‐29. Brit J Pharmacol. 1995;116:2866–2872. doi:10.1111/j.1476-5381.1995.tb15938.x. 8680718 PMC1909228

[51] Kolios G, Rooney N, Murphy C, Robertson D, Westwick J. Expression of inducible nitric oxide synthase activity in human colon epithelial cells: modulation by T lymphocyte derived cytokines. Gut. 1998;43:56–63. doi:10.1136/gut.43.1.56. 9771406 PMC1727175

[52] Khan KNM, Paulson SK, Verburg KM, Lefkowith JB, Maziasz TJ. Pharmacology of cyclooxygenase-2 inhibition in the kidney. Kidney Int. 2002;61:1210–1219. doi:10.1046/j.1523-1755.2002.00263.x. 11918727

[53] Riendeau D, Charleson S, Cromlish W, Mancini JA, Wong E, Guay J. Comparison of the cyclooxygenase-1 inhibitory properties of nonsteroidal anti-inflammatory drugs and selective COX-2 inhibitors, using sensitive microsomal and platelet assays. Can J Physiol Pharmacol. 1997;75:1088–1095. doi:10.1139/y97-130. 9365818

[54] Yang G, Lee K, Lee M, Ham I, Choi H-Y. Inhibition of lipopolysaccharide-induced nitric oxide and prostaglandin E_2_ production by chloroform fraction of *Cudrania tricuspidata* in RAW 264.7 macrophages. BMC Complement Altern Med. 2012;12:250. doi:10.1186/1472-6882-12-250. 23228109 PMC3575384

[55] Fábrega M-J, Rodríguez-Nogales A, Garrido-Mesa J, Algieri F, Badía J, Giménez R, Gálvez J, Baldomà L. Intestinal anti-inflammatory effects of outer membrane vesicles from *Escherichia coli* Nissle 1917 in DSS-experimental colitis in mice. Front Microbiol. 2017;8:1274. doi:10.3389/fmicb.2017.01274. 28744268 PMC5504144

[56] Madara J, Pappenheimer J. Structural basis for physiological regulation of paracellular pathways in intestinal epithelia. J Membr Biol. 1987;100:149–164. doi:10.1007/BF02209147. 3430571

[57] Clayburgh DR, Barrett TA, Tang Y, Meddings JB, Van Eldik LJ, Watterson DM, Clarke LL, Mrsny RJ, Turner JR. Epithelial myosin light chain kinase–dependent barrier dysfunction mediates T cell activation–induced diarrhea in vivo. J Clin Invest. 2005;115:2702–2715. doi:10.1172/JCI24970. 16184195 PMC1224297

[58] Kawashima R, Kawakami F, Maekawa T, Yamamoto H, Koizumi W, Ichikawa T. Elemental diet moderates 5-fluorouracil-induced gastrointestinal mucositis through mucus barrier alteration. Cancer Chemother Pharmacol. 2015;76:269–277. doi:10.1007/s00280-015-2790-z. 26048344

[59] Wrzosek L, Miquel S, Noordine M-L, Bouet S, Chevalier-Curt MJ, Robert V, Philippe C, Bridonneau C, Cherbuy C, Robbe-Masselot C. *Bacteroides thetaiotaomicron* and *Faecalibacterium prausnitzii* influence the production of mucus glycans and the development of goblet cells in the colonic epithelium of a gnotobiotic model rodent. BMC Biol. 2013;11:61. doi:10.1186/1741-7007-11-61. 23692866 PMC3673873

[60] Fishman JE, Levy G, Alli V, Sheth S, Lu Q, Deitch EA. Oxidative modification of the intestinal mucus layer is a critical but unrecognized component of trauma hemorrhagic shock-induced gut barrier failure. Am J Physiol Gastrointest Liver Physiol. 2013;304:G57–G63. doi:10.1152/ajpgi.00170.2012. 23125158 PMC3543631

[61] Silva SD, Robbe-Masselot C, Ait-Belgnaoui A, Mancuso A, Mercade-Loubière M, Salvador-Cartier C, Gillet M, Ferrier L, Loubière P, Dague E. Stress disrupts intestinal mucus barrier in rats via mucin O-glycosylation shift: prevention by a probiotic treatment. Am J Physiol Gastrointest Liver Physiol. 2014;307:G420–G9. doi:10.1152/ajpgi.00290.2013. 24970779

[62] Hasnain SZ, Tauro S, Das I, Tong H, Chen ACH, Jeffery PL, McDonald V, Florin TH, McGuckin MA. IL-10 promotes production of intestinal mucus by suppressing protein misfolding and endoplasmic reticulum stress in goblet cells. Gastroenterology. 2013;144:357–68. e9. doi:10.1053/j.gastro.2012.10.043. 23123183

[63] Burger-van Paassen N, Van der Sluis M, Bouma J, Korteland-van Male AM, Lu P, Van Seuningen I, Boehm G, van Goudoever JB, Renes IB. Colitis development during the suckling-weaning transition in mucin Muc2-deficient mice. Am J Physiol Gastrointest Liver Physiol. 2011;301:G667–G78. doi:10.1152/ajpgi.00199.2010. 21700902

[64] Kouznetsova I, Peitz U, Vieth M, Meyer F, Vestergaard EM, Malfertheiner P, Roessner A, Lippert H, Hoffmann W. A gradient of TFF3 (trefoil factor family 3) peptide synthesis within the normal human gastric mucosa. Cell Tissue Res. 2004;316:155–165. doi:10.1007/s00441-004-0854-1. 14968359

[65] Furuse M, Hirase T, Itoh M, Nagafuchi A, Yonemura S, Tsukita S, Tsukita S. Occludin: a novel integral membrane protein localizing at tight junctions. J Cell Biol. 1993;123:1777–1788. doi:10.1083/jcb.123.6.1777. 8276896 PMC2290891

[66] Stevenson BR, Siliciano JD, Mooseker MS, Goodenough DA. Identification of ZO-1: a high molecular weight polypeptide associated with the tight junction (zonula occludens) in a variety of epithelia. J Cell Biol. 1986;103:755–766. doi:10.1083/jcb.103.3.755. 3528172 PMC2114282

[67] Beatch M, Jesaitis LA, Gallin WJ, Goodenough DA, Stevenson BR. The tight junction protein ZO-2 contains three PDZ (PSD-95/Discs-Large/ZO-1) domains and an alternatively spliced region. J Biol Chem. 1996;271:25723–25726. doi:10.1074/jbc.271.42.25723. 8824195

[68] Citi S, Sabanay H, Jakes R, Geiger B, Kendrick-Jones J. Cingulin, a new peripheral component of tight junctions. Nature. 1988;333:272–276. doi:10.1038/333272a0. 3285223

[69] D’atri F, Citi S. Cingulin interacts with F‐actin in vitro. FEBS Lett. 2001;507:21–24. doi:10.1016/S0014-5793(01)02936-2. 11682052

[70] Wang F, Graham WV, Wang Y, Witkowski ED, Schwarz BT, Turner JR. Interferon-gamma and tumor necrosis factor-alpha synergize to induce intestinal epithelial barrier dysfunction by up-regulating myosin light chain kinase expression. Am J Pathol. 2005;166:409–419. doi:10.1016/S0002-9440(10)62264-X. 15681825 PMC1237049

[71] Al-Sadi R, Ye D, Dokladny K, Ma TY. Mechanism of IL-1beta-induced increase in intestinal epithelial tight junction permeability. J Immunol. 2008;180:5653–5661. doi:10.4049/jimmunol.180.8.5653. 18390750 PMC3035485

[72] Blair SA, Kane SV, Clayburgh DR, Turner JR. Epithelial myosin light chain kinase expression and activity are upregulated in inflammatory bowel disease. Lab Invest. 2006;86:191–201. doi:10.1038/labinvest.3700373. 16402035

[73] Stillwell W. Chapter 24 - cell death, apoptosis. In: Stillwell W, editor. An introduction to biological membranes. 2nd ed. Cambridge (MA): Elsevier; 2016. p. 539–546.

[74] Lowe SW, Lin AW. Apoptosis in cancer. Carcinogenesis. 2000;21:485–495. doi:10.1093/carcin/21.3.485. 10688869

[75] Toshiyuki M, Reed JC. Tumor suppressor p53 is a direct transcriptional activator of the human bax gene. Cell. 1995;80:293–299. doi:10.1016/0092-8674(95)90412-3. 7834749

[76] Finucane DM, Bossy-Wetzel E, Waterhouse NJ, Cotter TG, Green DR. Bax-induced caspase activation and apoptosis via cytochrome c release from mitochondria is inhibitable by Bcl-xL. J Biol Chem. 1999;274:2225–2233. doi:10.1074/jbc.274.4.2225. 9890985

[77] Hockenbery D, Nuñez G, Milliman C, Schreiber RD, Korsmeyer SJ. Bcl-2 is an inner mitochondrial membrane protein that blocks programmed cell death. Nature. 1990;348:334. doi:10.1038/348334a0. 2250705

[78] Park H-A, Licznerski P, Mnatsakanyan N, Niu Y, Sacchetti S, Wu J, Polster BM, Alavian KN, Jonas EA. Inhibition of Bcl-xL prevents pro-death actions of ΔN-Bcl-xL at the mitochondrial inner membrane during glutamate excitotoxicity. Cell Death Differ. 2017;24:1963–1974. doi:10.1038/cdd.2017.123. 28777375 PMC5635221

[79] Satyanarayana A, Hilton MB, Kaldis P. p21 Inhibits Cdk1 in the absence of Cdk2 to maintain the G1/S phase DNA damage checkpoint. Mol Biol Cell. 2008;19:65–77. doi:10.1091/mbc.e07-06-0525. 17942597 PMC2174178

[80] Brantley DM, Chen C-L, Muraoka RS, Bushdid PB, Bradberry JL, Kittrell F, Medina D, Matrisian LM, Kerr LD, Yull FE. Nuclear factor-κB (NF-κB) regulates proliferation and branching in mouse mammary epithelium. Mol Biol Cell. 2001;12:1445–1455. doi:10.1091/mbc.12.5.1445. 11359934 PMC34596

[81] Oeckinghaus A, Ghosh S. The NF-κB family of transcription factors and its regulation. Cold Spring Harb Perspect Biol. 2009;1:a000034. doi:10.1101/cshperspect.a000034. 20066092 PMC2773619

[82] Baeuerle PA, Baltimore D. I kappa B: a specific inhibitor of the NF-kappa B transcription factor. Science. 1988;242:540–546. doi:10.1126/science.3140380. 3140380

[83] Li Vivian SW, Ng Ser S, Boersema Paul J, Low Teck Y, Karthaus Wouter R, Gerlach Jan P, Mohammed S, Heck Albert JR, Maurice Madelon M, Mahmoudi T, et al. Wnt Signaling through inhibition of β-catenin degradation in an intact Axin1 complex. Cell. 2012;149:1245–1256. doi:10.1016/j.cell.2012.05.002. 22682247

[84] Markiewicz E, Tilgner K, Barker N, van de Wetering M, Clevers H, Dorobek M, Hausmanowa-Petrusewicz I, Ramaekers FCS, Broers JLV, Blankesteijn WM, et al. The inner nuclear membrane protein emerin regulates beta-catenin activity by restricting its accumulation in the nucleus. Embo J. 2006;25:3275–3285. doi:10.1038/sj.emboj.7601230. 16858403 PMC1523183

[85] Kobayashi M, Honma T, Matsuda Y, Suzuki Y, Narisawa R, Ajioka Y, Asakura H. Nuclear translocation of beta-catenin in colorectal cancer. Br J Cancer. 2000;82:1689–1693. doi:10.1054/bjoc.1999.1112. 10817505 PMC2374509

[86] Voronkov A, Krauss S. Wnt/beta-catenin signaling and small molecule inhibitors. Curr Pharm Des. 2013;19:634–664. doi:10.2174/138161213804581837. 23016862 PMC3529405

[87] Peters BA, Dominianni C, Shapiro JA, Church TR, Wu J, Miller G, Yuen E, Freiman H, Lustbader I, Salik J. The gut microbiota in conventional and serrated precursors of colorectal cancer. Microbiome. 2016;4:69. doi:10.1186/s40168-016-0218-6. 28038683 PMC5203720

[88] Claesson MJ, Jeffery IB, Conde S, Power SE, O’connor EM, Cusack S, Harris HM, Coakley M, Lakshminarayanan B, O’Sullivan O. Gut microbiota composition correlates with diet and health in the elderly. Nature. 2012;488:178–184. doi:10.1038/nature11319. 22797518

[89] Henao-Mejia J, Elinav E, Jin C, Hao L, Mehal WZ, Strowig T, Thaiss CA, Kau AL, Eisenbarth SC, Jurczak MJ. Inflammasome-mediated dysbiosis regulates progression of NAFLD and obesity. Nature. 2012;482:179–185. doi:10.1038/nature10809. 22297845 PMC3276682

[90] Huang G, Khan I, Li X, Chen L, Leong W, Ho LT, Hsiao WW. Ginsenosides Rb3 and Rd reduce polyps formation while reinstate the dysbiotic gut microbiota and the intestinal microenvironment in Apc Min/+ mice. Sci Rep. 2017;7:12552. doi:10.1038/s41598-017-12644-5. 28970547 PMC5624945

[91] Kigerl KA, Hall JC, Wang L, Mo X, Yu Z, Popovich PG. Gut dysbiosis impairs recovery after spinal cord injury. J Exp Med. 2016;213:2603–2620. doi:10.1084/jem.20151345. 27810921 PMC5110012

[92] Hong Y-S, Ahn Y-T, Park J-C, Lee J-H, Lee H, Huh C-S, Kim D-H, Ryu DH, Hwang G-S. 1 H NMR-based metabonomic assessment of probiotic effects in a colitis mouse model. Arch Pharm Res. 2010;33:1091–1101. doi:10.1007/s12272-010-0716-1. 20661720

[93] Livak KJ, Schmittgen TD. Analysis of relative gene expression data using real-time quantitative PCR and the 2^− ΔΔCT^ method. Methods. 2001;25:402–408. doi:10.1006/meth.2001.1262. 11846609

[94] Lane D. 16S/23S rRNA sequencing. In: Stackebrandt E, Goodfellow M editors. Nucleic acid techniques in bacterial systematics. Hoboken (NJ): John Wiley and Sons; 1991. p. 115–175.

[95] Kim O-S, Cho Y-J, Lee K, Yoon S-H, Kim M, Na H, Park S-C, Jeon YS, Lee J-H, Yi H. Introducing EzTaxon-e: a prokaryotic 16S rRNA gene sequence database with phylotypes that represent uncultured species. Int J Sys Evol Microbiol. 2012;62:716–721. doi:10.1099/ijs.0.038075-0. 22140171

[96] Caporaso JG, Kuczynski J, Stombaugh J, Bittinger K, Bushman FD, Costello EK, Fierer N, Pena AG, Goodrich JK, Gordon JI. QIIME allows analysis of high-throughput community sequencing data. Nat Methods. 2010;7:335. doi:10.1038/nmeth.f.303. 20383131 PMC3156573

[97] Hoving LR, Heijink M, van Harmelen V, van Dijk KW, Giera M. GC-MS analysis of short-chain fatty acids in feces, cecum content, and blood samples. In: Giera M editor. Clinical metabolomics. methods in molecular biology. New York (NY): Springer; 2018. p. 247–256. 10.1007/978-1-4939-7592-1_17 29363078

