# Supplementary material for: Cancer-protective effect of a synbiotic combination between Lactobacillus gasseri 505 and a Cudrania tricuspidata leaf extract on colitis-associated colorectal cancer
Source: Gut Microbes. 2020 Jul 14;12(1):1785803. doi: 10.1080/19490976.2020.1785803 (PMC7524312; doi:10.1080/19490976.2020.1785803)
Supplement: Supplemental Material [file KGMI_A_1785803_SM1375.docx]

**Supplementary Information for**

**Cancer-protective effect of a synbiotic combination between *Lactobacillus gasseri* 505 and a *Cudrania tricuspidata* leaf extract on colitis-associated colorectal cancer**

**Nam Su Oh^a,†^, Ji Young Lee^b,†, ‡^, You-Tae Kim^c,†^, Sae Hun Kim^b^, and Ju-Hoon Lee^c^**

*^a^ Department of Food and Biotechnology, Korea University, Sejong 30019, South Korea*

*^b^ Department of Biotechnology, College of Life Sciences and Biotechnology, Korea University, Seoul 02841, South Korea*

*^c^ Department of Food Science and Biotechnology, Graduate School of Biotechnology, Kyung Hee University, Yongin 17104, South Korea*

**Corresponding authors: Sae Hun Kim and Ju-Hoon Lee**

Email: [saehkim@korea.ac.kr](mailto:saehkim@korea.ac.kr), [juhlee@khu.ac.kr](mailto:juhlee@khu.ac.kr)

**This file includes:**

Figure S1, Tables S1 and S2

**Figure S1.** Tumorigenesis analysis using (A) the ratio of colon weight versus colon length, (B) the number of tumor cells per mouse, and (C) hyperplasia score. The ratio of colon weight versus colon length were gradually reduced and the lowest in FCT, suggesting that FCT has efficiently recovery and protection activities against shortening of AOM-DSS-treated colon (Figure S1(a)). In addition to Figure 1(e), formation of tumor cells were extensively observed using counting of tumer cell numbers per mouse and its diameter determination, showing that the number of tumor cells as well as the number of ≥3 diameter large tumor cells were reduced (Figure S1(b)), suggesting that AOM/DSS mice revealed notable progress in high-grade macroadenoma and advanced adenocarcinoma but tumor formation was efficiently supressed by FCT. The hyperplasia score showed that AOM/DSS has the highest hyperplasia score, but the scores were gradually reduced in LG, CT, and FCT. As shown in Figure S1(c), the AOM/DSS group showed dysplastic epithelium. However, CT and FCT significantly mitigated these symptoms in the colonic tissue of AOM/DSS-induced CAC mice. In particular, the hyperplasia score in the FCT group was a 2.7-fold decrease as compared to that in the AOM/DSS group. The hyperplasia scores were estimated by the method of Jin et al.^1^


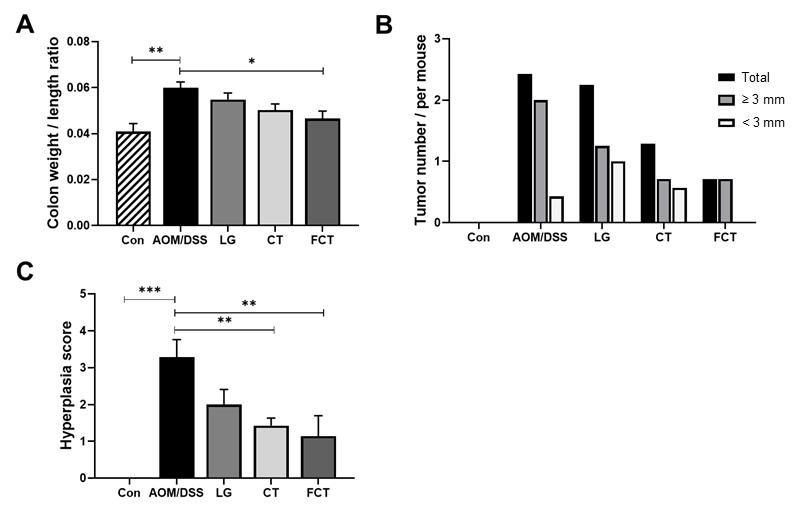


1. Jin BR, Chung KS, Lee M, An HJ. High-fat diet propelled AOM/DSS-induced colitis-associated colon cancer alleviated by administration of *Aster glehni* via STAT3 signaling pathway. Biology. 2020;9(2) 24

**Table S1.** Antibodies used in this study.

| Antibody | Supplier | Cat. No. | Host | Dilution |
| --- | --- | --- | --- | --- |
| iNOS | Santa cruz | SC-651 | Rabbit | 1:100 |
| COX-2 | Santa cruz | SC-7951 | Rabbit | 1:100 |
| p21 | Santa cruz | SC-397 | Rabbit | 1:100 |
| p53 | Cell Signaling Technology | 2524 | Mouse | 1:500 |
| Bcl-2 | Santa cruz | SC-7382 | Mouse | 1:100 |
| Bcl-xL | Cell Signaling Technology | 2764 | Rabbit | 1:500 |
| Bax | Cell Signaling Technology | 2772 | Rabbit | 1:500 |
| MUC2 | Santa cruz | SC-13312 | Goat | 1:100 |
| Occludin | Abcam | ab167161 | Rabbit | 1:500 |
| ZO-1 | Abcam | ab61357 | Mouse | 1:500 |
| TFF3 | Thermo Fisher Scientific | PA5-21081 | Rabbit | 1:100 |
| β-catenin | Cell Signaling Technology | 8480 | Rabbit | 1:200 |
| NF-κB | Cell Signaling Technology | 8242 | Rabbit | 1:500 |
| IκB-α | Cell Signaling Technology | 4812 | Rabbit | 1:500 |
| β-actin | Cell Signaling Technology | 4970 | Rabbit | 1:1000 |

**Table S2.** Real-time PCR primer sequences used in this study.

| Primer | Sequence (5'→ 3') | | Annealing Temp. |
| --- | --- | --- | --- |
| TNF-α | Forward | CTGAACTTCGGGGTGATCGG | 62.0°C |
|  | Reverse | GGCTTGTCACTCGAATTTTGAGA |  |
| IFN-γ | Forward | AGCCCTATTACAGCACAG | 56.5°C |
|  | Reverse | TTCTAACAACAAGTATCCC |  |
| IL-1β | Forward | CAACCAACAAGTGATATTCTCCATG | 57.0°C |
|  | Reverse | GATCCACACTCTCCAGCTGCA |  |
| IL-6 | Forward | AAGTCGGAGGCTTAATTACACATGT | 56.0°C |
|  | Reverse | CCATTGCACAACTCTTTTCTCATTC |  |
| IL-4 | Forward | GGTCTCAACCCCCAGCTAGT | 61.5°C |
|  | Reverse | GCCGATGATCTCTCTCAAGTGAT |  |
| IL-10 | Forward | CTTACTGACTGGCATGAGGATCA | 60.5°C |
|  | Reverse | GCAGCTCTAGGAGCATGTGG |  |
| iNOS | Forward | CCCTTCCGAAGTTTCTGGCAGCAGC | 64.5°C |
|  | Reverse | GGCTGTCAGAGAGCCTCGTGGCTTTGG |  |
| COX-2 | Forward | GAAGTCTTTGGTCTGGTGCCT | 58.5°C |
|  | Reverse | GCTCCTGCTTGAGTATGTCG |  |
| p21 | Forward | CCTGGTGATGTCCGACCTG | 61.5°C |
|  | Reverse | CCATGAGCGCATCGCAATC |  |
| p53 | Forward | CCCCTGTCATCTTTTGTCCCT | 60.5°C |
|  | Reverse | AGCTGGCAGAATAGCTTATTGAG |  |
| Bcl-2 | Forward | GCTACCGTCGTGACTTCGC | 60.5°C |
|  | Reverse | CCCCACCGAACTCAAAGAAGG |  |
| Bcl-xL | Forward | GGCACTGTGCGTGGAAAGCGTA | 62.0°C |
|  | Reverse | CCGCCGTTCTCCTGGATCCA |  |
| Bax | Forward | AGACAGGGGCCTTTTTGCTAC | 61.5°C |
|  | Reverse | AATTCGCCGGAGACACTCG |  |
| MUC2 | Forward | ATGCCCACCTCCTCAAAGAC | 55.5°C |
|  | Reverse | GTAGTTTCCGTTGGAACAGTGAA |  |
| Occludin | Forward | TCTGCTTCATCGCTTCCTTAG | 56.0°C |
|  | Reverse | GTCGGGTTCACTCCCATTA |  |
| ZO-1 | Forward | AGGACACCAAAGCATGTGAG | 53.5°C |
|  | Reverse | GGCATTCCTGCTGGTTACA |  |
| TFF3 | Forward | TAATGCTGTTGGTGGTCCTG | 63.5°C |
|  | Reverse | CAGCCACGGTTGTTACACTG |  |
| β-catenin | Forward | TCTCCTTGGCTGGCCTTTCTA | 58.0°C |
|  | Reverse | GTCACACAGCCCTGTCAAGA |  |
| GAPDH | Forward | GACGGCCGCATCTTCTTGT | 58.5°C |
|  | Reverse | CAGTGCCAGCCTCGTCCCGTACAA |  |
